# Assessment of nutrient adequacy and associated factors among lactating women of rural Bangladesh using observed intake: Findings from Bangladesh Integrated Household Survey 2018–2019

**DOI:** 10.1002/fsn3.3044

**Published:** 2022-09-15

**Authors:** Saiful Islam, Ahmed Jubayer, Md. Moniruzzaman Nayan, Md. Hafizul Islam, Abira Nowar

**Affiliations:** ^1^ Institute of Nutrition and Food Science University of Dhaka Dhaka Bangladesh; ^2^ Bangladesh Institute of Social Research (BISR) Trust Dhaka Bangladesh; ^3^ National Heart Foundation Dhaka Bangladesh

**Keywords:** Bangladesh, dietary intakes, lactating women, mean adequacy ratio, nutrient adequacy

## Abstract

Lactating women of low‐ and middle‐income countries are prone to develop deficiencies in essential nutrients due to various demographic and socioeconomic components. This study aimed to estimate the adequacy of dietary intake and the determinants of diet quality of lactating women in rural Bangladesh. One‐day dietary recall (24‐Hour recall) data of 973 lactating women were obtained from the Bangladesh Integrated Household Survey (BIHS) 2018–2019. Nutrient adequacy was determined using the Estimated Average Requirement (EAR) cut‐point approach. The molar ratios of phytate to zinc, calcium, and iron were calculated. Mean Adequacy Ratio (MAR) was calculated to measure diet quality, and multiple linear regression analysis was performed to assess the sociodemographic determinants of MAR. While the intakes of protein and carbohydrate were equal to/above the Acceptable Macronutrient Distribution Range (AMDR) among most of the subjects, intakes of total energy and fat were below the reference intakes for 74.4% and 98.3%, respectively. Nutrient adequacy remained unmet for riboflavin, calcium, vitamin A, and folate among most (87.2%–97.6%) of the study population, and the mean (*SD*) MAR was 0.72 (0.12). Cereals were the major contributor of energy and B vitamins, while protein and iron mainly came from plant‐based sources. The molar ratio of phytate to iron was greater than the critical limit among most respondents. Maternal Body Mass Index (BMI) (beta = 0.003, *p* = .014) and education level (beta = 0.017, *p* = .038) were associated with their diet quality. The diet of most lactating women in rural Bangladesh indicates the inadequacy of several micronutrients. This can lead to a worsening of the double burden of malnutrition in women. Lactating women should be given special consideration when designing food and nutrition programs for reproductive women in rural Bangladesh.

## Graphical abstract

Our study focused exclusively on adequacy of nutrient intake and the determinants of overall diet quality among lactating women of rural Bangladesh from a population‐weighted, nationally representative large sample. The diet of most lactating women of rural Bangladesh indicates the inadequacy of several micronutrients especially riboflavin, calcium, vitamin A, and folic acid. Lactating women should be given special consideration when designing food and nutrition programs among reproductive women in rural Bangladesh.

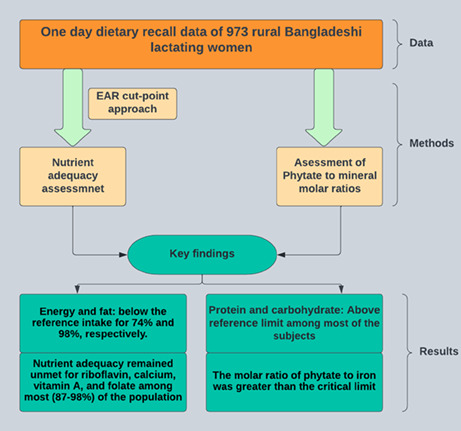

## 1 INTRODUCTION

During the lactating stage, women start producing a secretion called “breast milk” that contributes immensely to the sustainability of the offspring. Breastfeeding is considered to be a major determining factor of child growth and development since it is the sole source of nourishment for the child before initiation of the complementary feeding (Segura et al., 2016). The mother's diet and nutrient storage have a significant impact on the composition and yield of breast milk as do other factors (Segura et al., 2016). The content in breast milk of fat‐ and water‐soluble vitamins such as thiamin, riboflavin, vitamin A, vitamin B_6_, vitamin D, vitamin B_12_, and minerals like selenium and iodine is especially reduced in case of maternal deficiency of respective nutrients (Allen, 2012; Azizi & Smyth, 2009). This makes the lactation period one of the most nutritionally demanding stages of maternal life.

To meet the caloric expenditure during the mother's milk production process and the transfer of nutrients while breastfeeding, requirements of energy and micronutrients are comparatively higher during the lactation period than those of adulthood and even pregnancy (Sserwanja et al., 2021). Consequently, lactating women require around 500 additional kilocalories (kcal) per day in comparison to the recommendation for women of childbearing age (Joint FAO/WHO/UNU Expert Consultation, 2005). Breastfeeding also increases the need for some micronutrients, including vitamin A, riboflavin, vitamin B_6_, and vitamin C, by as much as 50% or more (Segura et al., 2016). As the micronutrient concentration in breastmilk depends entirely on the mother's nutrition and micronutrient status, a deficit of nutrient‐dense foods in the maternal diet may result in a high risk of suboptimal micronutrient status among exclusively breastfed infants (Allen & Dror, 2018).

Maternal and child malnutrition is one of the major public health concerns in Bangladesh, with a high maternal prevalence of anemia (Rahman et al., 2021). Bangladesh National Micronutrient Status Survey (International Centre for Diarrhoeal Disease Research, Bangladesh – Icddr, B 2013) and Ahmed et al. (2016) reported inadequacy of micronutrient intake and biochemical deficiencies of vitamin A, iron, and zinc among reproductive‐age women of rural Bangladesh. Nonetheless, little is known about what women eat during the lactation period. Few studies have investigated the diet quality and dietary diversity among the reproductive‐age women in Bangladesh (Akter et al., 2021; Grandner et al., 2021; Sununtnasuk & Fiedler, 2017). However, majority of the results are not disaggregated for pregnant and lactating women and suffer from inadequate power due to limited sample size. A recently published study (Grandner et al., 2021) reported dietary nutrient intakes by lactating women but the results are limited to a few nutrients based on more than a decade‐old survey in rural Bangladesh. Furthermore, the study did not analyze the factors associated with lower‐than‐recommended nutrient intake among lactating women. With the paucity of nationally representative up‐to‐date dietary data on lactating women, limited programmatic guidance can be provided when a nutritional intervention/program specifically targets the lactating women as direct beneficiaries.

Identification of suitable predictors of nutrient intake can contribute to developing appropriate intervention strategies for the improvement of maternal and child nutrition. During the breastfeeding period, the demand for micronutrients increases. However, a limited dietary diversity, plant‐based monotonous dietary practices, and lack of availability and affordability of nutrient‐dense foods provoke women in low‐ and middle‐income countries to be prone to suffer from micronutrient deficiencies, also known as hidden hunger. Poor hygiene and repeated infections also play an important role in the mother's micronutrient status (Henjum et al., 2015). Moreover, poor nutrition knowledge, cultural taboos, and gender inequity in the intrahousehold food distribution aggravate poor nutrient intake and maternal malnutrition (Beyene et al., 2021). A study recently showed that factors such as age, marital status, educational level, and monthly household income affected the overall diet quality of reproductive‐age women of rural Bangladesh (Akter et al., 2021).

In comparison to the women living in urban areas, the rural women of Bangladesh enjoy less decision‐making power, inadequate access to health care, and suffer from various types of social injustice (Shannon et al., 2008). Poverty coupled with social beliefs, cultural practices, and lack of education is so prevalent that these factors not only hamper women's reproductive health but also affect health of their children (Harding et al., 2017). Thus, assessing the nutrient adequacy of rural women and identifying the suitable predictors of nutrient intake can contribute to developing context‐specific intervention strategies for the improvement of their maternal health and child nutrition.

A nationally representative rural survey data were used in the present study to identify the factors impacting the individual nutrient intake of lactating women. The study aimed to measure the overall diet quality of lactating women of rural Bangladesh by quantifying the adequacy level of the relevant micro‐ and macronutrients by using their observed intake. Furthermore, the study extends previous findings by demonstrating key food groups contributing to nutrient intake and calculating intake ratios between different dietary minerals. Finally, the association between different sociodemographic variables and nutrient intake was studied as well.

## 2 METHODS

### 2.1 Data source and sampling

The current study used data from the third round of the Bangladesh Integrated Household Survey (BIHS) conducted between 2018 and 2019 (International Food Policy Research Institute – IFPRI, 2020). The BIHS was conducted under the supervision of the IFPRI with the funding from the United States Agency for International Development (USAID). BIHSs provide high‐quality data for evidence‐based policy research to address specific food security and agricultural development issues in Bangladesh. To date, BIHS is the most comprehensive and nationally representative household survey in rural Bangladesh, providing detailed data on agricultural production and practice, dietary intake and anthropometric information of individual household members, and data related to women empowerment. The BIHS sample is representative of rural Bangladesh as well as rural areas in each of the country's seven administrative divisions: Barisal, Chittagong, Dhaka, Khulna, Rajshahi, Rangpur, and Sylhet. A total of 5605 rural households were sampled in this household‐level survey using a two‐stage stratified sampling (Ahmed et al., 2013).

### 2.2 Data collection

The BIHS questionnaire was designed to collect useful data in an integrated format to answer a variety of research questions. The draft questionnaire prepared by IFPRI researchers was reviewed by USAID, officials of the Food Planning and Monitoring Unit (FPMU) of the Ministry of Food, researchers, and other stakeholders in Bangladesh. In addition, based on the review of the previous two surveys, the final questionnaire included extra questions for the third round of the BIHS.

Under the supervision and guidance of senior IFPRI researchers, Data Analysis and Technical Assistance (DATA) Limited implemented the BIHS. IFPRI researchers and DATA experts trained the survey enumerators and supervisors, mostly having a master's degree in social science, nutrition, or home economics. In the field, a printed version of the questionnaire was utilized to gather data, and weight and height scales (digital Health O' Meter weighing scales) were used for anthropometric measurements. An adult male household member (generally the household head) was interviewed by a male interviewer, and an adult female household member was interviewed by a female interviewer (typically the wife of the head of the household) (Ahmed et al., 2013).

Detailed data on 24‐Hour (24‐HR) dietary recall of all members of the household were collected, as this approach is validated for low‐ and middle‐income countries (Gibson et al., 2017). Individual‐level food consumption data were collected using multiple‐pass, 24‐HR recall, and food‐weighing methods for meals prepared at home. The female household member who held the responsibility for preparing and serving meals was interviewed to record recipes and the weight of raw ingredients of food items prepared during the previous 24 h. The quantity (cooked weight) of each dish consumed by each household member was also reported by that female member (Ahmed et al., 2013).

### 2.3 Sample selection

The present study focused on the dietary information of rural lactating women. Only women who answered “yes” to the question “Are you lactating?” were included in the present analyses. Lactating women, for whom detailed dietary information was missing, were excluded. Lastly, subjects with implausible calorie consumption for at‐home meals in rural Bangladeshi families, defined as greater than 4205 kcal/day, were eliminated. Sununtnasuk and Fiedler (2017) have recently estimated this value as the upper bound of the 95% confidence interval (CI) for the mean per capita calorie consumption for rural Bangladeshi adults. Our final study sample consisted of 973 lactating women.

### 2.4 Dietary data analysis

The following information was gathered for each member in the household: the names of the foods/mixed dishes consumed by the individual, as well as the weight of each food/dish consumed. Additionally, the quantity of salt used in cooking was noted. Therefore, the amount of sodium consumed was determined by the amount of sodium found in food and the amount of salt used in cooking. The following equation was employed to determine the equivalent of raw ingredients consumed by an individual (lactating women in the present analysis) of the family.

(1) $$\;\mathrm{Raw}\;\text{weight of ingredients}\;\left(\text{individual}\right)=\frac{\mathrm{raw}\;\text{weight of ingredients}\;\left(\text{household}\right)\times \text{cooked weight of consumed foods}/\text{mixed dishes}\;\left(\text{individual}\right)}{\text{cooked weight of foods}/\text{mixed dishes}\;\left(\text{household}\right).}$$

The nutrient content of the extracted data of lactating women was estimated using the Food Composition Tables for Bangladesh (FCTB) (Shaheen et al., 2022) and the Indian Food Composition Tables (IFCT) (Longvah et al., 2017). In the case of the foods that were not available on the local food composition table, the IFCT was followed. For the phytate content of foods unavailable in both local and IFCT, the Global food composition database for phytate (PhyFoodComp 1.0) was used (FAO/IZiNCG, 2018).

A probability approach could not be applied for the nature of BIHS data. The external within‐person variance estimates method—an alternative way to adjust nutrient intake distribution could not be followed as well, since similar dietary survey data in the local context were not available. Hence, the nutrient adequacy of lactating women was identified by comparing observed individual nutrient intake data with the requirements recommended by the Indian Council of Medical Research (ICMR) and National Institute of Nutrition (NIN) (ICMR_NIN expert group, 2020) using the EAR cut‐off approach.

Total Energy Expenditure (TEE) was used to calculate total energy requirements by multiplying an individual's Basal metabolic rate (BMR) by their Physical activity level (PAL). An extra 500 kcal was added to recover the cost of lactation. BMR was assessed using an individual's body weight and computed using the Joint FAO/WHO/UNU Expert Consultation group (2005) prediction algorithms based on sex and age. The individual's reported primary employment was used to determine their PAL and associated values (which were then classified as light (1.4) or moderate (1.7), or heavy (2)) (Joint FAO/WHO/UNU Expert Consultation, 2005). Using the data from the first round of the BIHS, Sununtnasuk and Fiedler (2017) also followed the same approach to calculate TEE for Bangladeshi adults.

Acceptable Macronutrient Distribution Range (AMDR) was calculated as the percentage of Energy (E%) for protein, carbohydrate, and fat. For all micronutrients (vitamin A, vitamin C, thiamin, riboflavin, niacin, folic acid, iron, zinc, calcium, phosphorus, sodium, potassium, and magnesium), individual intake data were compared against the corresponding Estimated Average Requirement (EAR) value (ICMR_NIN expert group, 2020). Since there is no EAR for fiber, the Recommended Dietary Allowance (RDA) value was considered to analyze this macromolecule (ICMR_NIN expert group, 2020). In addition, the Nutrient Adequacy Ratio (NAR)—the ratio between the subject's actual intake and reference intake—was calculated for each nutrient (Equation 2) (Hatløy et al., 1998). Following a previous study (Akter et al., 2021), the Mean Adequacy Ratio (MAR) was calculated using the NAR values of 16 nutrients (protein, carbohydrate, fat, dietary fiber, vitamin A, vitamin C, thiamin, riboflavin, niacin, folic acid, iron, zinc, calcium, phosphorus, potassium, magnesium) as an overall measure of diet quality. NAR values were truncated at 1 to prevent the compensation of a nutrient with NAR greater than 1 for a nutrient with a lower NAR. Then according to Equation 3, MAR was calculated by averaging all NAR values.

The MAR was reported on a scale from 0 to 1, with 0 indicating that the requirement for no nutrients was met and 1 indicating that the requirements for all nutrients were met.

(2) $$\mathrm{NAR}=\frac{\text{Daily nutrient intake}}{\text{Reference intake}}$$

(3) $$\mathrm{MAR}=\frac{\sum \mathrm{NAR}\left(\text{each truncated}\;\mathrm{at}\;1\right)}{\text{Number of nutrients}}$$

All the food items were categorized into five food groups (cereals; nuts and legumes; fruits and vegetables; meat, fish, and eggs; milk and milk products) according to the FCTB and the amount of individual nutrient intake contributed by each food group was assessed.

The molar ratios of phytate to zinc, calcium, and iron were calculated as the millimoles of phytate intake per day divided by the millimoles of zinc, iron, or calcium intake, respectively (Ma et al., 2007). The millimoles of phytic acid, zinc, calcium, and iron were calculated by dividing the milligrams of phytic acid by 660 (molecular weight of phytic acid), the milligrams of zinc by 65.38 (atomic weight of zinc), the milligrams of calcium by 40.078 (atomic weight of calcium), and the milligrams of iron by 55.845 (atomic weight of iron). The proportion of lactating women with ratios above the suggested critical values was calculated using the following values: phytate: calcium > 0.24 (Morris & Ellis, 1989), phytate: iron > 1 (Hallberg et al., 1987), phytate: zinc > 15 (Turnlund et al., 1984), and phytate × calcium/zinc > 200 (Bindra et al., 1986).

### 2.5 Covariates

A set of individual and household‐level factors were used to reduce possible bias which may have influenced the diet. Based on relevant research, these factors were selected a priori (Arsenault et al., 2013; Grandner et al., 2021; Sununtnasuk & Fiedler, 2017). Individual‐level factors of lactating women included age, Body mass index (BMI), educational level, decision‐making power in household food expenditure, and income‐earning status. The Asian‐specific BMI cut‐offs were used to measure the nutritional status of lactating women (WHO expert consultation, 2004). Household‐level factors included food security status, monthly per capita food expenditure, livestock ownership, and family size. The Food Insecurity Experience Scale (FIES), an experience‐based food insecurity experience scale, was used to gauge household food security, and the production of any livestock, poultry, or fish over the previous year was considered to evaluate livestock ownership status.

### 2.6 Statistical analysis

Statistical Package for Social Science (SPSS), version 25 was used to perform all statistical analyses. Normal distribution of variables was investigated before analysis through visual scrutiny of the statistical plot (i.e., histogram, Q–Q (quantile–quantile) plot, detrended Q–Q plot, Box plot) and by using statistical test—Kolmogorov–Smirnov and Shapiro–Wilk tests (Peat & Barton, 2014). An exploratory assessment was conducted to identify the determinants of nutrient intake with multiple linear regression analysis, including all possible covariates. The stepwise forward entry method was used to select those variables that were significant (*p* < .05) in simple linear regression analyses for inclusion in the multiple linear regression model. Statistical significance was defined as a *p*‐value less than .05 in two‐tailed tests.

The underlying assumption of linear regression model building was tested before analysis. Variance Inflation Factor (VIF) was examined to check the amount of multicollinearity in the model, and VIF greater than 2 was considered to indicate multicollinearity (Peat & Barton, 2014). The residual for each case was calculated, and the normality of residuals was inspected through the residual plot as well as other normality check procedures, such as descriptive statistics, normality plot, and statistical test. Cook's distance and leverage values were observed to identify multivariate outliers. The critical value for Cook's distance was set to 1, which was 0.05 for leverage value.

### 2.7 Ethics statement

The Ministry of Agriculture of Bangladesh reviewed the questionnaire and authorized the BIHS 2018–2019. Oral consent to participate in the survey was taken from the respondents. Ethical clearance from the BIHS authorities is not necessary for access to BIHS datasets.

## 3 RESULTS

### 3.1 Characteristics of the study sample

Table 1 provides a snapshot of the general demographic information of the study population. The mean (*SD*) age of the lactating women was 26.51 (6.31) years, and about 19.5% of them were at early age (≤20 years). The mean (*SD*) BMI was found to be 21.89 (3.7) kg/m^2^, with just 13.5% having decision‐making capability. The majority of study participants (93%) were housewives, and over 45% lived in food‐insecure families.

**TABLE 1:** Characteristics of lactating women participating in Bangladesh Integrated Household Survey (BIHS) 2018–2019 (*N* = 973)

Background characteristics	*n* (%)
Age (in years)	
≤ 20	190 (19.5)
21–25	251 (25.8)
26–30	276 (28.4)
31–35	175 (18.0)
>35	81 (8.3)
Body Mass Index, Mean (*SD*)	21.89 (3.7)
Nutritional status	
Underweight (BMI: <18.5 kg/m^2^)	185 (19.0)
Normal (BMI: 18.5–24.9 kg/m^2^)	585 (60.1)
Overweight (BMI: 25–29.99 kg/m^2^)	163 (16.7)
Obese (BMI: ≥30 kg/m^2^)	26 (2.7)
Missing	14 (1.4)
Education level	
Never attended school	114 (11.7)
Primary or below	305 (31.5)
Secondary or higher	554 (56.9)
Residence	
Barisal	77 (7.9)
Chittagong	159 (16.3)
Dhaka	284 (29.2)
Khulna	71 (7.3)
Rajshahi	105 (10.8)
Rangpur	112 (11.5)
Sylhet	165 (17.0)
Household food security	
Food secure	536 (55.1)
Food insecure	437 (44.9)
Involvement in economic activity	
Yes	68 (6.99)
No	905 (93.01)
Decision‐making capacity of women	
Yes	131 (13.5)
No	842 (86.5)
Household production of livestock	
Yes	506 (52.0)
No	467 (48.0)
Family size	
<4	331 (34.0)
5–6	452 (46.5)
≥7	190 (19.5)

### 3.2 Intake and adherence to dietary recommendations for energy and macronutrients

The daily energy and macronutrient intake of the evaluated participants and the percentage of lactating women below, above, or equal to the AMDR, RDA, and TEE are presented in Table 2. The median energy intake was 2250.33 kcal/day and 74.4% of the population's energy intake was below their TEE. For macronutrients, the intake was compared with the AMDR, and it was found that the carbohydrate intake was above AMDR (45%–65% of TEE) in all individuals. The median NAR of carbohydrate was 1.73. Around 99.9% of the population had protein intake above the AMDR and the median NAR (2.47) was the highest for protein. However, 98.3% of the lactating womens' fat intake was below the acceptable range (20%–35% of TEE). Also, more than one‐third (35%) of the lactating women consumed dietary fiber below the RDA (30–38 g/day).

**TABLE 2:** Energy and macronutrients' intake among Bangladeshi rural lactating women (*N* = 973)

		Intake/ person/day			Percentage of subjects below reference intake		
Macronutrient	Reference intake range	Median	Q_25_	Q_75_	Below reference intake	Above /equal to reference intake	Median NAR
Energy (kcal/day)^a^		2250.33	1892.89	2594.33	74.4	25.6	0.85
Carbohydrate (g/day)	‐	424.77	361.67	502.11	‐	‐	‐
Carbohydrate (E%)^b^	45–65	78.14	74.03	81.06	0.00	100	1.73
Protein (g/day)	‐	67.84	54.77	84.40	‐	‐	‐
Protein (E%)^b^	5–15	12.1	10.4	13.85	0.1	99.9	2.47
Total fat (g/day)	‐	15.7	11.03	22.25	‐	‐	‐
Total fat (E%)^c^	20–35	6.52	4.62	8.83	98.3	1.7	0.78
Dietary fiber (g/day)	30	25.55	17.38	33.1	65.0	35.0	0.85

Abbreviation: E%, Percentage of total energy intake; NAR, Nutrient Adequacy Ratio = actual intake/ recommended intake.

^a^ Total Energy Expenditure (TEE) was calculated by multiplying an individual's Basal metabolic rate (BMR) by their Physical activity level (PAL); NAR was calculated as the ratio of Energy intake to TEE; intake of below TEE and equal/above TEE.

^b^ E% of carbohydrate and protein was calculated as carbohydrate or protein intakes in gram×4 × 100/total energy intake in kcal;

^c^ E% of total fat was calculated as the total fat intake in gram×9 × 100/total energy intake in kcal.

### 3.3 Adherence to micronutrient recommendations

Table 3 summarizes the participants' estimated daily micronutrient intake compared to the EAR. The proportion of lactating women in rural Bangladesh with nutrient intake below EAR varied between the nutrients. A total of 49.5% and 63% of the lactating women consumed zinc and iron below their EAR, respectively. Riboflavin (97.6%) followed by calcium (93%), vitamin A (89.1%), and folic acid (87.2%) were the micronutrients in which the percentage of intake below the EAR was the highest. Niacin had the highest median NAR (1.81) among all micronutrients. The mean (*SD*) MAR of all 16 nutrients was 0.72 (0.12).

**TABLE 3:** Daily dietary micronutrients' intake and prevalence of inadequate intake among rural lactating women (*N* = 973)

	Intake/ person/day				Percentage with intakes		
Micronutrient	Median	Q_25_	Q_75_	EAR	Below EAR^b^	Median NAR	MAR^a^
Vitamin A (μg/day)	93.69	46.34	239.92	720	89.1	0.13	0.72
Vitamin C (mg/day)	75.49	45.47	131.37	95	62.1	0.79	
Thiamin (mg/day)	1.56	1.20	2.01	1.7	58.3	0.91	
Riboflavin (mg/day)	0.84	0.63	1.14	2.5	97.6	0.33	
Niacin (mg/day)	29.03	22.86	36.21	16	6.3	1.81	
Folic acid (μg/day)	158.92	116.79	218.94	280	87.2	0.56	
Iron (mg/day)	14.08	10.71	18.47	16	63.0	0.88	
Zinc (mg/day)	12.07	9.94	14.4	11.8	49.5	1.0	
Calcium (mg/day)	351.45	231.53	596.22	1000	93.0	0.35	
Phosphorus (mg/day)	1115.3	836.69	1431.16	1000	38.2	1.11	
Sodium (mg/day)	1434.66	233.87	4052.5	2000	55.7	0.72	
Potassium (mg/day)	2731.53	2105.62	3549.03	3500	74.4	0.78	
Magnesium (mg/day)	372.83	266.51	480.19	335	44.6	1.05	

Abbreviations: EAR, Estimated Average Requirement; NAR, Nutrient Adequacy Ratio = actual intake/ recommended intake; MAR, Mean Adequacy Ratio.

^a^ MAR calculation: NAR values were truncated at 1 with NAR greater than/equal to 1 for a nutrient and at 0 with NAR below 1. Then according to equation 2, MAR was calculated by averaging all NAR values, except sodium.

^b^ EAR for Bangladeshi lactating women; EAR for all micronutrients were taken from the ICMR_NIN expert group (2020).

### 3.4 Contribution of food groups to daily nutrient intake

Table 4 depicts the contribution of food groups to daily energy and nutrient intake. More than three‐fourths (76.73%) of their daily energy came from cereal‐based foods. In the case of protein, they mainly consumed plant‐based protein (69.85%) rather than high‐quality protein. All the B vitamins were obtained mainly from cereal‐based foods except folate, which mainly came from fruits and vegetables (56.43%). The majority of vitamin C (97.4%) and vitamin A (56.4%) came from the intake of fruits and vegetables. Only 4.8% of total dietary iron came from animal sources and the greater portion of consumed iron came from cereals (35.27%). Among the bone minerals, calcium came from both fruits, vegetables, and animal sources, whereas the greatest portion of magnesium and phosphorous came from the cereal group (53% and 46%, respectively).

**TABLE 4:** Percentage of energy, protein, and micronutrients from different food groups

	Cereal	Nuts and legumes	Fruits and vegetables	Meat, fish, and eggs	Dairy
Energy	76.73	6.55	7.68	4.5	0.63
Protein	44.39	15.37	8.42	29.23	0.93
Vitamin A	0.0	0.35	54.77	36.67	2.48
Vitamin C	0.0	0.0	97.38	0.06	0.35
Thiamin	53.99	16.65	12.81	11.89	0.72
Riboflavin	31.75	14.89	30.41	9.72	5.71
Niacin	64.51	9.07	7.79	15.87	0.14
Folate	27.71	1.72	56.43	5.21	0.88
Iron	35.27	22.93	19.96	4.81	0.14
Zinc	52.92	15.81	16.57	8.97	0.83
Calcium	11.39	14.52	24.29	29.88	5.22
Magnesium	52.93	15.55	21.32	5.81	0.68
Phosphorus	46.22	18.33	11.54	18.26	1.77
Potassium	19.36	16.16	27.69	7.53	1.03

### 3.5 Relative intake ratios

Mean (*SD*) of sodium/potassium, calcium/phosphorus, and phosphorus/protein ratios were found to be 0.86 (0.84), 0.42 (0.31), and 16.79 (6.54), respectively. Median molar ratios of phytate to iron, zinc, calcium, and phytate × calcium/zinc were 6.12, 8.11, 0.15, and 67.56, respectively. Phytate:iron molar ratio was greater than the suggested critical limit (1.0) for 98.9% of the respondents (Table 5), while only 2.7% of them had a phytate:zinc molar ratio above the cut‐off point (15). In contrast to phytate:iron and phytate:calcium molar ratios, the phytate × calcium/zinc molar ratio of 8.2% of the subjects was higher than the reference limit.

**TABLE 5:** Molar ratios of dietary phytate to iron, zinc, calcium, and phytate×calcium/zinc among rural lactating women of Bangladesh (*N* = 973)

	Reference limit	Median (IQR)	% below the cut‐off	% above the cut‐off
Phytate:iron	1	6.12 (4.01, 7.74)	1.1	98.9
Phytate:zinc	15	8.11 (6.11, 10.19)	97.3	2.7
Phytate:calcium	0.24	0.15 (0.09, 0.26)	71.7	28.3
(Phytate×calcium)/zinc	200	67.56 (42.01, 125.12)	91.8	8.2

### 3.6 Factors associated with nutrients' intake

A multivariate linear regression model was fitted to examine the effects of different maternal and socioeconomic characteristics on nutrient intake (Table 6). After adjusting with potential covariates, education level and BMI of lactating women were found to have a significant association with overall diet quality measured as MAR. For each unit increment in BMI, MAR would increase by 0.003 units (beta = 0.003, *p* = .014). Lactating women who had secondary or higher‐level education had a better‐quality diet than those who had up to primary‐level education (beta = 0.017, *p* = .038).

**TABLE 6:** Factors associated with the diet quality of lactating women (*N* = 973)

Parameter	Coefficient (95% CI)	*p*‐value
Body Mass Index (BMI)	0.003 (0.001, 0.005)	.014
Education level		
Secondary or higher Primary or below (reference)	0.017 (0.001, 0.034)	.038

*Note*: Adjusted for the age of lactating women, decision‐making power, income‐earning status, household food security, monthly per capita food expenditure, livestock ownership, and family size.

## 4 DISCUSSION

The current study assessed the diet quality of lactating women of rural Bangladesh in terms of nutrient adequacy and estimated the contribution of major food groups toward the intake of 17 different nutrients using secondary data from the BIHS 2018–2019. In our study, the energy intake of lactating women was found to be low compared to their requirements (Joint FAO/WHO/UNU Expert Consultation, 2005). Carbohydrate and protein intake was correspondingly high, whereas, total fat intake was inadequate. Our study findings confirmed rice and other cereal‐based food items as a major contributor to daily energy intake (almost 74%). Of the 13 micronutrients, the prevalence of inadequacy was particularly high (>87%) for four micronutrients: riboflavin, calcium, vitamin A, and folic acid. A low intake of micronutrient‐rich foods combined with increased nutrient requirements during the lactation period is probably the principal explanatory factor for the low adequacy of micronutrients. However, maternal BMI and education level were significantly associated with the overall diet quality of lactating women.

High inadequacy of vitamin A, riboflavin, folate, iron, and adequacy of niacin were consistent with findings of other studies conducted among pregnant and lactating women of Bangladesh (Arsenault et al., 2013; Nguyen et al., 2018). It is therefore surprising that the reported intake of zinc (12 mg/day) and vitamin C (75.5 mg/day) in our study was slightly higher than that documented in a prior study as 5.5 mg/day and 59 mg/day, respectively (Arsenault et al., 2013). It is to be noted that calcium intake was much lower than the EAR; however, the reported intake in the present study was more than double the quantity found in a study conducted in two rural subdistricts of Bangladesh (351.45 mg/day vs. 160 mg/day) by Arsenault et al. (2013). Poor dietary diversity with minimal consumption of milk and dairy products may account for insufficiency in calcium intake (Mirmiran et al., 2006). However, when the diet of a lactating woman does not provide sufficient calcium for breast‐milk production, the growth of the breastfeeding baby may be adversely affected and calcium would be released from the maternal skeleton, with possible short‐ or long‐term effects on their health (Kovacs, 2016). A study conducted in Iran found that the consumption of dairy products is positively correlated with the probability of calcium, phosphorus, zinc, and protein adequacy (Mirmiran et al., 2006). Therefore, educating lactating mothers to consume milk, cheese, and yogurt would help them ensure adequate calcium intake along with improved bone health.

We also observed a comparatively higher intake of phosphorus. Takeda et al. (2012) found diets high in phosphorus and low in calcium may interfere with calcium absorption and result in osteodystrophy. Similarly, the comparative calcium/phosphorus molar ratio may considerably affect bone health and is positively associated with lower bone mass (Lee et al., 2014). Given the findings of this study, it can be said that the low calcium/phosphorus might aggravate calcium deficiency in lactating women.

Although lactation does not necessitate an increase in iron requirements, we found that iron intake was woefully inadequate. This finding aligned with the findings of a higher risk of suboptimal iron status among reproductive‐aged women of Bangladesh found in another study (National Institute of Population Resaerch and Training – NIPORT, 2019). Lactating women get the majority of their iron from poorly bioavailable plant‐based foods, increasing their risk of anemia. Likewise, only about half of the women had adequate zinc intake, which is in line with previous research on zinc intake in women that indicates low biochemical zinc status among Non‐Pregnant Non‐Lactating (NPNL) and pregnant women of Bangladesh (Icddr, B 2013). Several factors may account for poor zinc status including poor bioavailability of zinc from the plant‐based diet of the Bangladeshi rural population.

One in nine NPNL women in Bangladesh has a folate deficiency (Icddr, B 2013). Our analysis suggested that very few women had adequate folate intake, despite the consumption of folate‐containing green leafy vegetables and lentils by the majority of the participants. There is a strong possibility that consumed quantities might be lower than reported; a situation that was beyond the scope of our analysis.

Moreover, the dietary sodium/potassium ratio was observed to be less than 1 (0.86 ± 0.84), reflecting that sodium intake was lower than potassium intake. Sodium and potassium are important for maintaining blood pressure and diets with low sodium and high potassium reduce the risk of hypertension and other cardiovascular diseases (Lee et al., 2013).

To measure the inhibitory effect of phytate on mineral bioavailability, the phytate to mineral molar ratios were calculated. Phytate is largely found in seeds, cereals, and pulses which is a storage form of phosphorus. Phytate:iron molar ratio of 1 or even above 0.4 impairs iron absorption significantly since the inhibitory effect is observed at a very low phytate concentration (2–10 mg/meal) (Gershfeld et al., 2011). The current study has supported the findings of a previous study that suggested very high phytate:iron molar ratio for Bangladeshi rural pregnant women most possibly due to plant‐based monotonous dietary practices (Hasan et al., 2016). In contrast, phytate:zinc molar ratio > 15 is likely to compromise zinc bioavailability (Morris & Ellis, 1989). The current study found only 2.7% of lactating women with phytate:zinc molar ratio >15, which was lower than that given in the previous study (12% among Bangladeshi rural pregnant women) (Hasan et al., 2016).

A phytate‐to‐calcium ratio of >0.24 is known to significantly decrease calcium absorption (Morris & Ellis, 1989). About one‐third (28.3%) of rural lactating women had phytate‐to‐calcium ratio above the suggested critical point which indicates the impairment of calcium absorption by phytate intake. A similar study among pregnant women of Bangladesh (Hasan et al., 2016) stated a higher proportion (52%) with phytate‐to‐calcium molar ratio exceeding the cut‐off value. However, to predict the synergistic effect of both calcium and phytate on zinc bioavailability, we calculated the molar ratio of (phytate × calcium) to zinc (Bindra et al., 1986; Gibson et al., 1991). Due to low calcium intake, the median (phytate × calcium)/zinc was only 67.56 and only 8.2% of women had a (phytate × calcium) to zinc ratio above the reference limit. Such low calcium intake also indicates that (phytate × calcium) to zinc ratio might not be a good indicator for predicting the interaction of phytate and calcium on zinc absorption (Ma et al., 2007).

In our analysis, we established a regression model to identify factors that are associated with the overall diet quality of the lactating mothers measured as MAR. Our analysis suggested a significant positive association between BMI and a higher level of education. However, in contrast to the present study, Akter et al. (2021) found a significant association for age and household monthly income. On the other hand, consistent with the present study, a recent publication in China (Zhao et al., 2016) reported the education level and BMI of lactating women as important predictors of the certain mineral consumption. A similar study among peri‐urban lactating women in Nepal (Henjum et al., 2015) also demonstrated that age, education level, and socioeconomic status were positively associated with nutrient adequacy.

## 5 THE IMPLICATION OF THE STUDY FINDINGS

Since nutrient requirements are different throughout maternal life, it is necessary to disaggregate dietary measures for different reproductive stages to avoid the risk of programmatic inaction. The current study focused exclusively on lactating women from a population‐weighted, nationally representative large sample which has important implications for the aptness and relevance of diet‐enhancing interventions. The results of this study provide supportive evidence for policymakers in developing food‐based dietary guidelines for lactating women in rural Bangladesh.

## 6 STRENGTH AND LIMITATIONS

One limitation of our data is its cross‐sectional nature providing only a snapshot that makes it impossible to establish a temporality of events or draw a causal inference. However, as suggested by Markovitz et al. (2012), cross‐sectional studies may be a better source of data for policy judgments in the public health community than longitudinal studies when risk factors vary more across space at a fixed moment in time than at a fixed location across time. Since this study was based on secondary data where dietary data were collected only at a single point of time (single 24‐HR recall method) that did not allow us to apply a probability approach to estimate usual intake distribution. In the absence of repeated dietary recall data in the local context, we could not employ external within‐person variance estimate method—an alternative approach to adjusting nutrient intake distribution. The potential risk of recall bias is another important limitation of the 24‐HR method where the exact amount of each food consumed cannot be investigated by enumerators. This poses a risk that our estimation may not fully reflect the real intake. Nevertheless, as the enumerators in this survey were well trained and the method is validated for low‐ and middle‐income countries (Gibson et al., 2017), it is expected to have fewer 24‐HR recall‐related limitations. Moreover, in some cases, we had to depend on the external food composition table (IFCT), but this may not be a great issue in the variation of nutrient contents of foods, as Bangladesh and India are in the same geographical region and quite a few similarities are observed in their food patterns. A major strength of our study was the robust sample size that ensures variability in dietary intake. Furthermore, a validated questionnaire was used for data collection.

## 7 CONCLUSION

Diets of lactating women could not ensure adequacy in several evaluated micronutrients, most notably riboflavin, calcium, vitamin A, and folic acid, while adequate intake was observed for certain nutrients including protein, carbohydrate, and niacin. The overall diet quality was significantly better in women with higher levels of education and BMI. In addition, the predominant source of almost all micronutrients was plant‐based foods that hinder well absorption of certain minerals. Appropriate intervention strategies may be needed to foster a healthier diet for lactating women in rural Bangladesh. Further studies are needed to replicate 24‐HR, so that the usual dietary pattern can be investigated.

## FUNDING INFORMATION

No funding was obtained to conduct this research.

## CONFLICT OF INTEREST

The authors declared no competing interest in any part of this article.

## Data Availability

BIHS datasets are available online at https://dataverse.harvard.edu/dataset.xhtml?persistentId=doi:10.7910/DVN/NXKLZJ
